# Uniform Noting for International Application of the Tumor-Stroma Ratio as an Easy Diagnostic Tool: Protocol for a Multicenter Prospective Cohort Study

**DOI:** 10.2196/13464

**Published:** 2019-06-14

**Authors:** Marloes Smit, Gabi van Pelt, Annet Roodvoets, Elma Meershoek-Klein Kranenbarg, Hein Putter, Rob Tollenaar, J Han van Krieken, Wilma Mesker

**Affiliations:** 1 Department of Surgery Leiden University Medical Center Leiden Netherlands; 2 Datacenter Surgery Leiden University Medical Center Leiden Netherlands; 3 Department of Medical Statistics Leiden University Medical Center Leiden Netherlands; 4 Department of Pathology Radboud University Medical Center Nijmegen Netherlands

**Keywords:** tumor-stroma ratio, colon cancer, pathology, observer variation, prospective study

## Abstract

**Background:**

Colon cancer treatment is dependent on the stage at diagnosis. The current Tumor-Node-Metastasis (TNM) staging for the selection of patients for adjuvant chemotherapy needs additional prognostic and predictive biomarkers. Better decision making for chemotherapy will result in reducing over- and undertreatment. We developed a new, easy-to-apply, practice-changing method to select colon cancer patients for adjuvant chemotherapy: the tumor-stroma ratio (TSR). The TSR distinguishes within stage II-III patients who will likely benefit from adjuvant chemotherapy and those who will not.

**Objective:**

The aim of the study was to add, in addition to the TNM classification, the TSR to current routine pathology evaluation. Pathologists will be instructed for scoring the TSR in combination with a quality assessment program. An international multicenter study will validate the parameter prospectively.

**Methods:**

The study is designed for future implementation of the TSR to the current TNM guidelines, using routinely Haematoxylin- and Eosin-stained tumor tissue sections. In part 1 of the study, an electronic learning (e-learning) module with a quality assessment program using the European Society of Pathology framework will be developed. This module will be used to assess the reliability and reproducibility of the TSR, conducted by national and international pathologists. Part 2 will involve the validation of the TSR in a prospective cohort of colon cancer p-stage II-III patients in a multicenter setting. In total, 1500 patients will be included.

**Results:**

The results of part 1 will be expected in the first half of 2019. For part 2, the inclusion of patients in the prospective study, which started at the end of 2018, will take 3 years with an additional follow-up after another 3 years.

**Conclusions:**

The main endpoints of this study are as follows: in part 1, trained (international) pathologists who are able to reliably score the TSR, resulting in low intra- and interobserver variation; in part 2, confirmation of significant survival differences for patients with a stroma-high tumor versus patients with a stroma-low tumor. On the basis of these findings, a modification in current treatment guidelines will be suggested.

**Trial Registration:**

Netherlands Trial Register NTR7270; https://www.trialregister.nl/trial/7072

**International Registered Report Identifier (IRRID):**

DERR1-10.2196/13464

## Introduction

### Background

Despite complete resection of the primary tumor and surrounding lymph nodes, colon cancer patients often develop recurrence of disease, caused by the remaining micrometastases. These can be treated with chemotherapy. However, as micrometastases are difficult to detect, treatment guidelines are usually based on tumor characteristics related to disease progression and survival, such as depth of invasion and lymph node metastasis. The current guidelines advise to give adjuvant chemotherapy to patients with stage III colon carcinoma and patients with stage II and one or more high-risk factors [1]. Only a part of the patients who are treated with chemotherapy will actually benefit. Furthermore, there is also substantial undertreatment because 25% of the stage II patients, who do not receive adjuvant chemotherapy, will develop recurrence or metastasis within 5 years [2]. Some patients with stage IIIA receive adjuvant chemotherapy, whereas in some cases, the prognosis is better compared with patients with stage IIB disease [1]. The selection of colon cancer patients for adjuvant treatment should be further improved to establish an optimal treatment regimen for each patient.

Over the last decade, the microenvironment or stromal (ie, nonepithelial) component of tumors has been studied intensively. There is increasing evidence that the tumor stroma plays an important role in the biological behavior of tumors, their growth, ability to metastasize, but also their response or resistance to anticancer drugs [3-6]. Tumors that are rich in stroma behave in a more aggressive way compared with tumors with little stroma [2,7].

### Tumor-Stroma Ratio

The tumor-stroma ratio (TSR) parameter is based on the amount of stroma within the primary tumor and can be determined, without extra costs, during routine pathology assessment. Using the TSR, stage II/III stroma-high (high-risk) patients can be adequately registered for treatment with chemotherapy, whereas for the (elderly) patients with stage III and stroma-low, further discussion is needed as to whether adjuvant therapy will benefit these patients. New guidelines for patient management will have consequences for better patient management leading to a more optimal selection for adjuvant chemotherapy with a potential reduction in costs.

A high stroma percentage (>50%) is an unfavorable prognostic factor. The TSR has been validated in various international studies with high interobserver agreements [2,7-12]. The TSR was discussed by the TNM Evaluation Committee, the Union for International Cancer Control, and the College of American Pathologists. They stated that our observations are important and novel and have the potential to be added to the TNM staging algorithm as prognosticator. They advocated validation in a prospective multicenter study and development of consensus agreement and a quality assessment program. This protocol elaborates on this recommendation.

### Objectives

The overall objective is the addition of the TSR to current routine pathology next to the TNM classification for clinical decision making.

Primary objective for each part of the project:

1. Part 1: To evaluate and improve the reliability and reproducibility of pathologists specifically instructed for TSR scoring.
2. Part 2: To confirm the prognostic power of the method to select patients at risk for the development of recurrence of disease resulting in high-level evidence for adaptation of guidelines.

## Methods

### Histopathological Scoring of the Tumor-Stroma Ratio

For the evaluation of the TSR, Haematoxylin and Eosin (H&E)–stained sections of the primary colon carcinoma, used in routine pathology to determine the T-stage (ie, the deepest part of the tumor), are analyzed using conventional microscopy. Areas with the largest amount of stroma are selected using a 2.5x or 5x objective. An area where both tumor and stromal tissue are present within this vision site is selected using a 10x objective. Tumor cells have to be present at all borders of the selected image field. Mucinous tumors, although more difficult, can also be scored; an area containing mucus may be used. However, the volume of mucus should be excluded when determining the TSR. Other challenging cases can be tumors with abundant necrosis and/or muscle tissue in between tumor glands. Necrotic areas or muscle tissue should be avoided in the scoring procedure. It is not necessary to score the TSR at the invasive front, picking a field with as much stroma as possible is more accurate.

**Figure 1:**
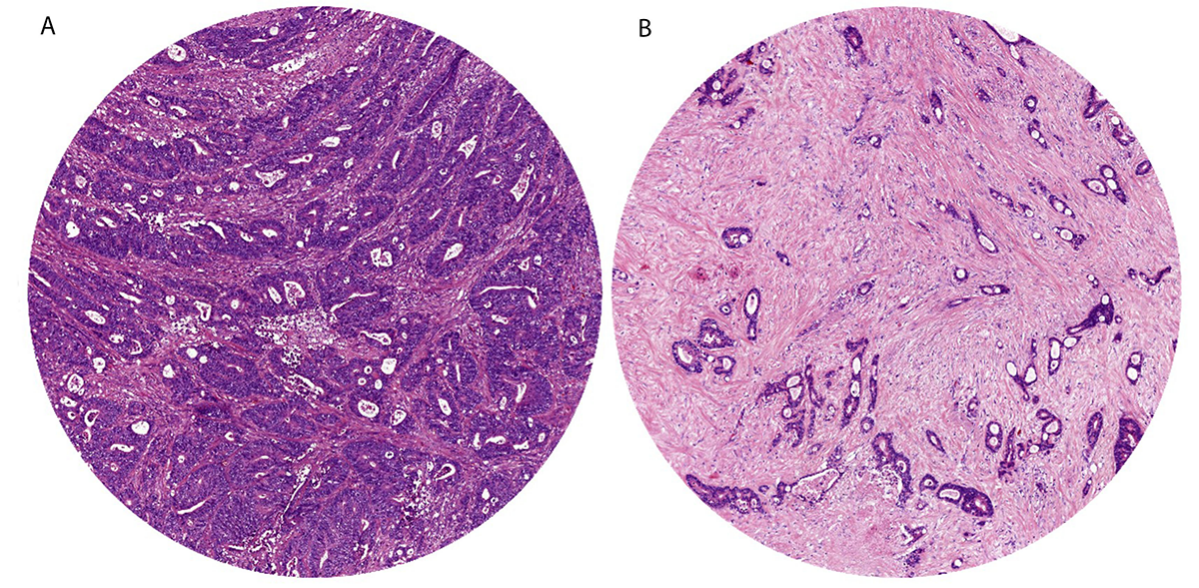
Examples of stroma-low colon cancer (A) and stroma-high colon cancer (B).

Stroma-high is defined as >50% stromal area and stroma-low, as ≤50% stromal area in the histological section (Figure 1). This cut-off has been determined a priori with maximum discriminative power [2,7,9]. Even if there is only one image field with a stroma-high score, this image field is decisive to categorize the patient as stroma-high.

The scoring protocol is available in an instruction movie on the Uniform Noting for International application of the Tumor-stroma ratio as Easy Diagnostic tool (UNITED) study website [13] and in written form published by our group [14].

### Study Design

Part 1 will consist of an electronic learning (e-learning) module which has been developed with a quality assessment program in the framework of the European Society of Pathology (ESP) External Quality Assessment program. Using this module, a reliability and reproducibility study on H&E-stained tumor tissues will be conducted among national and international pathologists.

Part 2 will involve validation in a prospective cohort of colon cancer stage II-III patients within this multicenter setting. The inclusion is expected to take 3 years, with a 3-year follow-up period.

### Patient Description

In the UNITED study, all patients are diagnosed with pathological stage (p-stage) II or p-stage III colon cancer. For e-learning, H&E-stained slides of stage II-III colon cancer patients were selected in a retrospective manner. Material was obtained from the archive of the Department of Pathology of the Leiden University Medical Center (LUMC).

### Part 1: The E-Learning Module

**Figure 2:**
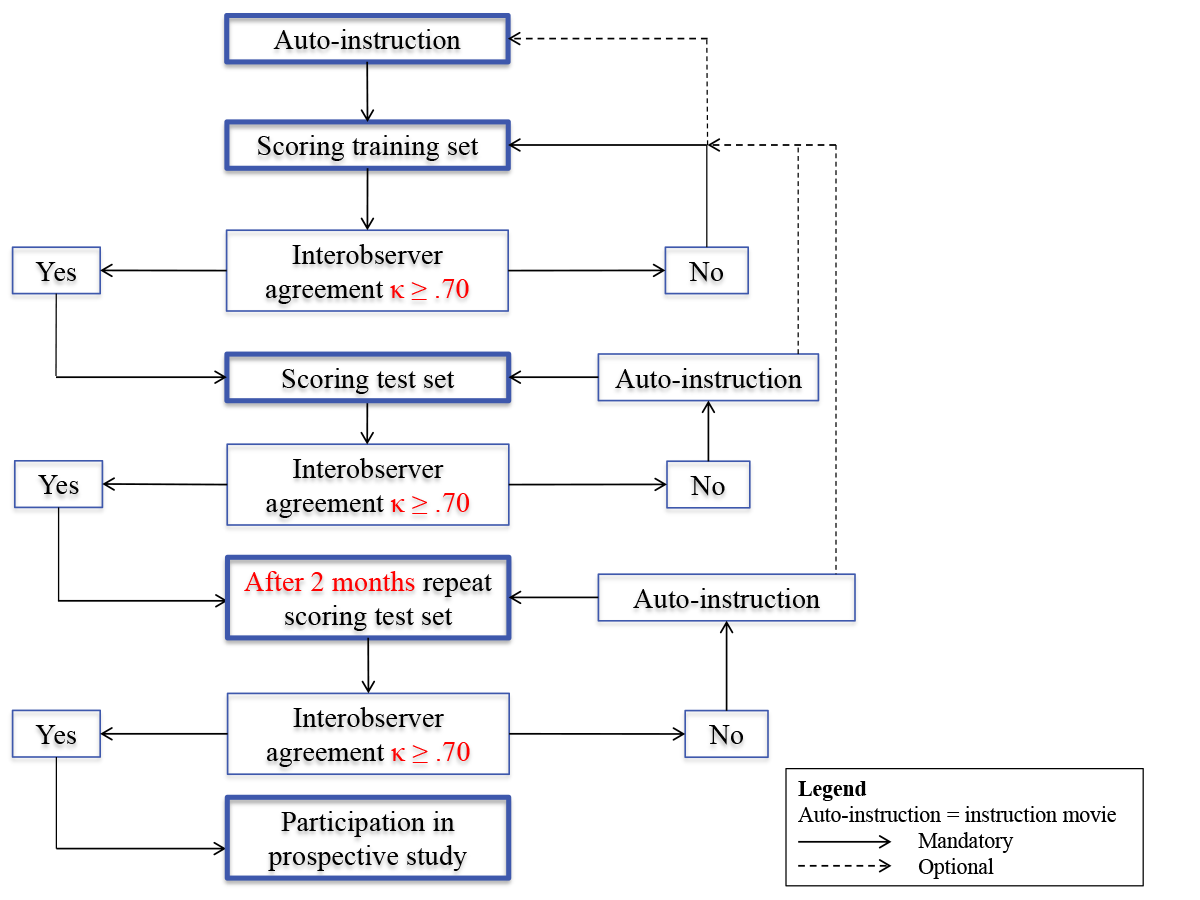
Flowchart for the instruction of participating pathologists using the e-learning module.

An e-learning module has been developed in the framework of the ESP. The software used for the e-learning is PathXL Tutor version 6.1.1.1. (Philips). This is a Web-based software that can be accessed worldwide. Participating pathologists receive specific user credentials for access to the e-learning sets. The workflow is shown in Figure 2 and includes an introduction film with the technical instructions. Hereafter, the participating pathologists may start the e-learning by analyzing the training set containing 40 cases.

TSR scores of participating pathologists will be compared with a reference score (consisting of 3 observers at the LUMC). If a pathologist does not pass a set (interobserver variability kappa<.70), he or she is asked to re-analyze the set. If need be, the instruction video and protocol can be studied again. If a pathologist passes the set (kappa≥.70), the pathologist is able to continue to the next set of 40 slides. The test set will be repeated after 2 months, thereafter inter- and intraobserver variability are determined. The pathologists are unaware of any clinical information or previous scoring.

The quality of TSR scoring by the participating pathologists will be monitored on a yearly basis by offering control series.

### Part 2: Validation of the Tumor-Stroma Ratio in a Prospective Study

After finishing the e-learning, the pathologist is well instructed to score TSR in the daily routine. To validate the TSR prospectively, a multicenter study is set up. The study aims to include, in the participating centers, a total of 1500 colon cancer patients who have undergone complete curative resection (R0 resection), independent of receiving adjuvant chemotherapy according to actual guidelines.

### Recruitment of Patients and Consent

Each consecutive eligible patient with a clinical stage I/II/III tumor will be informed about the study by their physician or research nurse. After informed consent, the pathologist is notified that the TSR can be determined. All patients, independent of gender and family history, are invited to participate. Medical history is no reason for exclusion, apart from malignancies within 10 years before the current colon carcinoma. Textboxes 1 and 2 describe the inclusion and exclusion criteria.

**Patient inclusion criteria.**

- Histologically proven colon carcinoma
- Complete curative resection (R0 resection)
- Clinical stage I (T1-2, N0, M0), II (T3-4, N0, M0) or III (any T, N1-2, M0)
- Aged ≥18 years
- Written informed consent

**Patient exclusion criteria.**

- Neo-adjuvant treatment; this influences the amount of tumor and stroma, by fibrosis formation
- Any malignancy within 10 years before the current colon carcinoma (except for basal cell carcinoma or cervical carcinoma in situ) or any colon carcinoma in history; owing to prolonged treatment or metastasis from earlier primary tumors that can influence the current colon carcinoma prognosis. Basal cell carcinoma and cervical carcinoma in situ do not have metastatic capacity
- Multiple synchronous colon tumors; patients with synchronous tumors are likely to have a worse prognosis and need a different approach for treatment
- Rectum tumors; these are defined as separate entities. Prognosis and treatment is different compared with colon tumors

Additional exclusion after surgery:

- p-stage I or stage IV; p-stage I is excluded as these patients will not receive adjuvant treatment. Stage IV patients are excluded as these patients are palliatively treated
- Deceased within 3 months after surgery; patients who die within 3 months after surgery die most often owing to comorbidity or surgical complications

### Safety Reporting and Risk Analysis

The patient material to be analyzed in this study is a conventional H&E-stained histological section of the primary tumor, obtained during the routine pathology process. The method is without any additional intervention and the study does not have consequences for the treatment of patients. Therefore, the safety or health of participating subjects will not be jeopardized in any way by this study. Consequently, no adverse events, serious adverse events, or suspected unexpected serious adverse events will occur owing to the study. A data safety monitoring board is not indicated.

### Data Storage

The LUMC Datacenter, Department of Surgery, is the Central Datacenter and responsible for supply of electronic Case Report Forms, study database, generation of queries within the database, and central monitoring.

Data will be stored in Castor Electronic Data Capture (Castor EDC; Castor, Amsterdam, the Netherlands) [15]. Castor EDC is a cloud-based electronic data capture platform, easy-to-use by researchers worldwide and highly secured. Data can be easily captured; therefore, data are of high quality and reusable. Data and documents will be stored for at least 15 years.

### Statistical Analysis

Statistical analysis will be performed using IBM SPSS Statistics version 25.0 in collaboration with the Department of Medical Statistics of the LUMC.

#### Part 1

For the analysis of the inter- and intraobserver variability, Cohen kappa coefficient will be used.

#### Part 2

##### Sample Size Calculation

For the prospective cohort, a sample size calculation has been performed for both stages based on earlier research findings [2,7].

- p-stage II patients: with a hazard ratio (HR) of 2.0, adjusted for TNM, and a known percentage of stroma-high patients in p-stage II of 20% to 25% [2,7], 114 recurrence events with 90% power are necessary. To achieve 114 recurrence events based on an event rate of 0.0575 per year (leading to a 5-year probability of 75% and 3-year recurrence probability of 84.2%), this leads to 722 patients.
- p-stage III patients: with an HR of 2.0, adjusted for TNM, and a known percentage of stroma-high patients in stage III of 30% to 35% [2,7], 97 recurrence events with 90% power are necessary. To achieve 97 recurrence events based on an event rate of 0.081 per year (leading to a 5-year probability of 66.7% and 3-year recurrence probability of 78.4%), this leads to 450 patients.

To obtain 1172 evaluable p-stage II/III, approximately 1500 (+25%) patients will be registered, as all p-stage I and stage IV patients will be excluded.

##### Statistical Analysis

Survival analysis will be performed using Kaplan-Meier survival analysis and differences in survival distributions will be tested using Log Rank statistics. The Cox proportional hazard model is used to determine the HR of explanatory variables for overall and disease-free survival (OS and DFS, respectively).

OS is defined as the time period between the date of surgery and the date of death from any cause or the date of the last follow-up. DFS is defined as the time between the date of surgery and the date of any recurrence (local, regional, or distant metastasis), date of new primary tumor, or date of death (any cause). If no event occurs, DFS is calculated as the time period until the date of last follow-up.

### Ethical Considerations

This project is registered with the Netherlands Trial Registry (NTR 7270). It will be conducted according to the Declaration of Helsinki, Forteza, Brazil, October 2013.

As this research plan uses existing H&E-stained sections, conventionally prepared for routine diagnostics, there is no risk for the patient, and we expect no problems with the regulatory authorities in the collaborating countries.

The UNITED study protocol has been approved by the Medical Research Ethics Committee (MREC) of the LUMC, study number p17.302. Before inclusion of patients in participating countries, the protocol will be endorsed by the MREC of each participating hospital.

Informed consent will be obtained from each eligible patient in oral and written form before surgery.

## Results

### Part 1

The e-learning started mid-2018 and the first results will be expected in the first half of 2019. The results will be presented within 6 to 12 months after the last pathologist has completed the e-learning module.

### Part 2

The first patients were included at the end of 2018. In total, 1500 patients are needed, and the expected inclusion time is about 3 years. A follow-up of 3 years is required. In late 2023, the first results are expected, and they will be presented within 12 to 18 months after the last follow-up.

## Discussion

The UNITED study has been developed to implement the TSR in routine pathology, in addition to the TNM classification and other known risk factors as an extra indicator for medical treatment decision making.

Earlier research validated the prognostic value of TSR in retrospective cohort studies. With the UNITED study, we aim to validate the prognostic value of the TSR in a prospective way.

The results of the e-learning will contribute to a standardized method and specifically trained pathologists. With the yearly quality assessments, the quality of the scoring method will be monitored and guaranteed.

Beside the tumor characteristics, as described in the TNM classification, to determine the p-stage, the microenvironment of the tumor is an important factor as well. The microenvironment of a tumor is a wide spread of different cell types. More tumor characteristics in the microenvironment are studied, such as tumor budding [16-21], Immunoscore [22-24], and desmoplastic reaction [19,25,26]. They are all independent prognostic biomarkers for survival [16-26]. Outside this protocol, we aim to study the relation between the different (microenvironment) biomarkers to better understand the role of the microenvironment and to further improve patient selection for adjuvant treatment.

Treatment decision making in oncology is a multidisciplinary process where medical oncologists play a pivotal role. These professionals will also be involved by the introduction of the TSR in daily clinical practice.

In conclusion, the UNITED study will, for the first time, evaluate the TSR in a prospective cohort to prove its prognostic value in stage II/III colon cancer. After completion of the UNITED study, the TSR will have the highest level of evidence for a prognostic marker and should be ready to use in the daily practice of all gastroenterology pathologists and also ready to play a role in clinical decision making.

## Abbreviations

Castor EDC: Castor Electronic Data Capture
DFS: disease-free survival
e-learning: electronic learning
ESP: European Society of Pathology
H&E: Haematoxylin and Eosin
HR: hazard ratio
LUMC: Leiden University Medical Center
MREC: Medical Research Ethics Committee
OS: overall survival
p-stage: pathological stage
TNM: Tumor-Node-Metastasis
TSR: tumor-stroma ratio
UNITED: Uniform Noting for International application of the Tumor-stroma ratio as Easy Diagnostic tool
